# A High-Sensitivity Sweat Glucose Biosensor Enabled by an In Situ Grown NiFe PBA on Porous Pt/Ni/Au-SPE

**DOI:** 10.3390/s26092908

**Published:** 2026-05-06

**Authors:** Huajie Shu, Qinglin Liu, Qianhui Wei, Changhui Mao, Feng Wei, Hailing Tu

**Affiliations:** 1 State Key Laboratory of Advanced Materials for Intelligent Sensing, GRINM Group Co., Ltd., Beijing 100088, China; 2 GRINM (Guangdong) Institute for Advanced Materials and Technology, Foshan 528000, China; 3 General Research Institute for Nonferrous Metals, Beijing 100088, China

**Keywords:** porous Pt/Ni, Prussian blue analogues, in situ synthesis, sweat glucose biosensor, electrochemical sensor

## Abstract

As a promising class of catalysts for enzymatic glucose sensors, Prussian blue analogues (PBAs) exhibit exceptional biomimetic activity. However, their performance is often constrained by poor intrinsic conductivity, which typically limits their sensitivity. To address this limitation, this study presents an effective approach using direct in situ growth of PBAs on the electrode substrates, which enables the effective integration of PBA-based electrochemical systems. A porous Ni framework was first electrodeposited onto a screen-printed gold electrode substrate, followed by the reduction of Pt onto the porous Ni. Subsequently, NiFe PBA was synthesized in situ using the porous Pt/Ni structure as a sacrificial template. Functionalized with glucose oxidase (GO_x_), the PBA/Pt/Ni biosensor exhibited excellent performance for glucose detection in buffer solution, with a high sensitivity of 262.6 μA mM^−1^·cm^−2^ and an ultra-low detection limit of 1.45 μM (calculated at a signal-to-noise ratio of 3, S/N = 3). Notably, its sensitivity corresponds to a two-fold enhancement relative to the electrodes modified with commercial Prussian blue using the conventional drop-casting method. Even when tested in human sweat samples, the biosensor achieved a high sensitivity of 236.4 μA mM^−1^·cm^−2^ and a linear detection range of 20–1000 μM, with the broad sensing range fully encompassing the typical physiological concentrations of glucose in human sweat. This excellent performance arises from the high specific surface area of the porous Pt/Ni structure and the tight connection between PBA and the sacrificial Ni anode. This research presents a promising design strategy for advanced, wearable, and non-invasive health-monitoring platforms.

## 1. Introduction

Diabetes remains a significant challenge in global health [1]. In order to relieve patients of suffering during glucose testing, non-invasive monitoring technology is being rapidly developed to replace the traditional invasive technology [2]. Sweat glucose sensing is considered to be a promising non-invasive route for indirectly detecting blood glucose levels [3,4]. However, the concentration of glucose in sweat is much lower than that in blood, which demands higher sensitivity and lower detection limits in sensors [5,6]. Increasing the specific surface area of electrode materials is an effective strategy for improving detection sensitivity [7,8,9]. The dynamic hydrogen bubble template (DHBT) electrodeposition method offers a viable route for fabricating porous materials [10,11]. Despite their demonstrated high sensitivity, noble metals (e.g., Pt, Pd, and Au) are challenging to process via DHBT, as they readily undergo electrodeposition at potentials near or above 0 V versus the reversible hydrogen electrode (RHE) [12,13]. Consequently, it is critical to develop the DHBT electrodeposition method to obtain porous Pt-based materials.

To enhance the sensitivity of enzymatic glucose sensing, Prussian blue (PB) and Prussian blue analogues (PBAs) represent an ideal class of co-catalysts for reducing the reaction potential and minimizing the interference from electroactive species in complex real-world samples [14,15,16,17]. Nevertheless, their poor intrinsic conductivity frequently compromises sensitivity, limiting their practical adoption. To address these kinetic limitations, various strategies have integrated PBAs with high-surface-area conductive scaffolds. For example, Thakur et al. developed porous carbon (PC)/PB composites to increase the active surface area [18]. However, the use of polymer binders in such composites often results in the partial blockage of catalytic sites and compromised electrical conductivity. More recently, a graphene aerogel/PB-based biosensing system leveraging a three-dimensional conductive network was reported to enhance electrochemical performance [19]. While these layered structures facilitate electron transport, their assembly often involves complex, multi-step procedures and yields stochastic interfacial contacts between PB and the graphene aerogel. Such discontinuous hetero-interfaces can still introduce local transport resistance and compromise mechanical robustness during continuous monitoring.

In parallel with enzymatic approaches, non-enzymatic glucose sensors have also seen remarkable progress in wearable formats. For example, a Janus fabric-based biosensor utilizing Cu_2_O as the sensing element achieved an ultrahigh sensitivity and demonstrated a fully integrated wireless early-warning system [20]. Similarly, Pt nanoparticles confined within phthalocyanine-based conductive metal–organic frameworks (Pc-MOFs) have been integrated into a microfluidic patch, enabling continuous 12-h sweat glucose monitoring with on-site pH and temperature calibration [21]. These non-enzymatic systems excel in sensitivity and operational stability by circumventing enzyme degradation. However, they are typically operate at relatively high anodic potentials, where electroactive interferents (e.g., ascorbic acid, uric acid) can be co-oxidized, necessitating complex differential measurements or selective membranes. In contrast, the enzymatic electrodes modified with PBAs operate at low cathodic potentials (∼−0.1 V vs. Ag/AgCl) for H_2_O_2_ reduction, which inherently minimizes interference from common sweat constituents. Therefore, the development of a well-structured, binder-free in situ synthesis strategy is essential—an approach that effectively prevents PBA particle agglomeration and minimizes adverse effects on material conductivity [22,23].

Herein, we report a novel three-step in situ strategy for constructing a high-performance wearable glucose biosensor. Utilizing the DHBT method, we first engineered a hierarchical micro/nano-porous nickel framework on a screen-printed gold electrode. Unlike traditional macro-porous metal foams or 3D carbon frameworks, this DHBT-derived Ni substrate provides a significantly higher electrochemically active surface area and active site density. Subsequently, a Pt film was electrodeposited, followed by the synthesis of a NiFe PBA layer via a sacrificial anode electrodeposition approach. Crucially, this in situ transformation establishes a robust chemical interface between the Ni substrate and the PBA layer, which is superior to the physical adsorption or heterogeneous nucleation typical of hydrothermal and chemical bath deposition methods. By minimizing interfacial resistance and preventing active material exfoliation, this integrated 3D architecture effectively optimizes electron transfer and mechanical durability, significantly improving the sensitivity and reliability of non-invasive glucose sensing in real sweat.

## 2. Experimental

### 2.1. Chemicals

Glucose (C_6_H_12_O_6_), single-walled carbon nanotubes (SWCNTs), potassium tetrachloroplatinate(II) (K_2_PtCl_4_), sulfuric acid (H_2_SO_4_), ammonium chloride (NH_4_Cl), nickel chloride (NiCl_2_), potassium ferricyanide (K_3_[Fe(CN)_6_]), hydrogen peroxide (H_2_O_2_), lactic acid (C_3_H_6_O_3_), uric acid (C_5_H_4_N_4_O_3_), sodium chloride (NaCl), potassium chloride (KCl), ascorbic acid (AA, C_6_H_8_O_6_), urea (CH_4_N_2_O), sucrose (C_12_H_22_O_11_), glucose oxidase (GO_x_), and phosphate-buffered saline (PBS) were purchased from Innochem Co., Ltd. (Beijing InnoChem Science & Technology Co., Ltd., Beijing, China). The Au screen-printed electrode (Au-SPE) was purchased from DropSens Metrohm (DRP 220AT) (Metrohm China Limited, Hongkong, China).

### 2.2. Synthesis of Porous Ni on Au-SPE Electrode

The Au-SPE was first cleaned using a 0.1 M H_2_SO_4_ solution via cyclic voltammetry (CV) at a potential range of 0 to 1.0 V (vs. Ag/AgCl) and at a scan rate of 100 mV/s repeated twice for 10 cycles each. Porous Ni structures were synthesized on the Au-SPE at a constant potential of −3 V (vs. Ag/AgCl) for 40 s in a solution containing 0.05 to 0.3 M NiCl_2_ and 2 M NH_4_Cl. For convenience, the obtained electrode was denoted as Ni.

### 2.3. Synthesis of Pt/Ni Electrode

The as-prepared Ni electrode was subsequently immersed in an electrolyte solution of 4 mM K_2_PtCl_4_ and 0.1 M H_2_SO_4_, and the synthesis of Pt nanoparticles was performed at a constant potential of −2 V (vs. Ag/AgCl) for 40 s.

### 2.4. Synthesis of PBA/Pt/Ni Electrode

To fabricate the PBA/Pt/Ni electrode, the Pt/Ni electrode was subjected to a sacrificial anode electrodeposition process by conducting 120 cyclic voltammetry cycles in an electrolyte comprising 1 mmol/L K_3_Fe(CN)_6_ and 1 mol/L Na_2_SO_4_. The scans were run at a rate of 100 mV/s over a potential range of 0 to 1 V (vs. Ag/AgCl). During this step, Ni dissolved from the porous Pt/Ni framework to supply the nickel ions, which subsequently reacted with ferricyanide ions in solution to form the NiFe Prussian blue analogues film on the surface of the electrode.

### 2.5. Immobilization of GO_x_@SWCNTs on Electrodes

Subsequently, 2 mg of SWCNTs were added to 1 mL PBS (pH 5.5) containing 0.1 wt% acetic acid and 1 wt% chitosan, and sonicated for 30 min. This solution was mixed with GO_x_/PBS solution in a volume ratio of 2:1 and sonicated for another 30 min. Then, 5 μL GO_x_ solution was drop-cast onto the as-prepared electrodes and stored for 12 h at 4 °C. Finally, 5 µL of Nafion/ethanol solution (1:10 *v*/*v*) was applied to the working electrode and allowed to dry for 30 min at room temperature.

### 2.6. Material Characterization

The X-ray diffraction (XRD) patterns were recorded on a Haoyuan DX-2700BH X-ray diffraction instrument (Dandong Haoyuan Instrument Co., Ltd., Dandong, China) with Cu Kα radiation. Scanning electron microscopy (SEM) tests were carried out on a Zeiss Crossbeam 350 instrument (Carl Zeiss Microscopy GmbH, Oberkochen, Germany) equipped with an Energy Dispersive Spectrometer (EDS) detector (Bruker XFlash 6160, Bruker Corporation, Billerica, MA, USA). The microstructure and composition were characterized by high-resolution transmission electron microscopy (TEM, JEOL JEM-2100F) (JEOL Ltd., Tokyo, Japan). X-ray photoelectron spectroscopy (XPS) spectra were detected using a Thermo Scientific K-Alpha instrument (Thermo Fisher Scientific, Waltham, MA, USA).

### 2.7. Electrochemical Measurements

All electrochemical measurements were carried out in a three-electrode system using the GAMRY Interface 1010E electrochemical analyzer (Gamry Instruments, Warminster, PA, USA), with screen-printed gold electrodes (Au-SPE, counter electrode: Au; reference electrode: Ag/AgCl; area of the working electrode: 0.11 cm^2^) as the base electrodes. Unless otherwise specified, PBS was used as the electrolyte. Cyclic voltammetry (CV) curves were collected at a scan rate of 100 mV s^−1^. The measurement of the electrochemical double layer capacitance (C_dl_) was performed under a range of voltages (0.1 to 0.2 V) with different scanning rates (10 to 50 mV s^−1^). All electrochemical performance metrics, including sensitivity, linear range, and limit of detection, were evaluated using three independently fabricated sensors (*n* = 3) to ensure statistical significance.

## 3. Results and Discussion

### 3.1. Characterization

**Figure 1:**
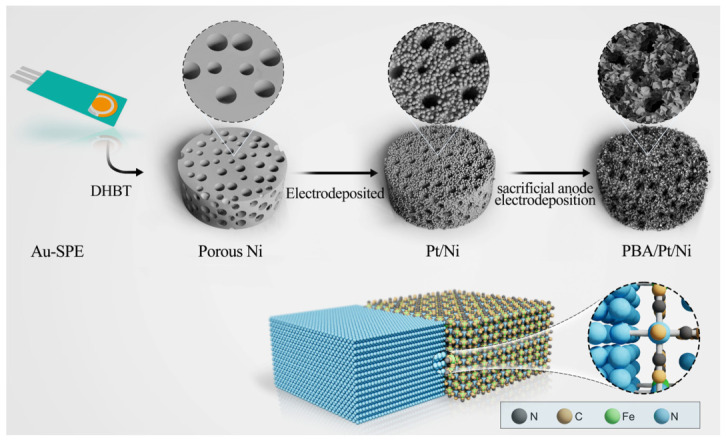
Schematic diagrams of the synthesis processes of PBA/Pt/Ni.

The fabrication process of the PBA/Pt/Ni biosensor electrode is schematically depicted in Figure 1. Initially, the nickel framework was fabricated onto an Au-SPE using the DHBT electrodeposition method. The morphology of the electrodeposition material was characterized by SEM. The pristine Au-SPE presented a rough planar morphology (Figure S1a). 

**Figure 2:**
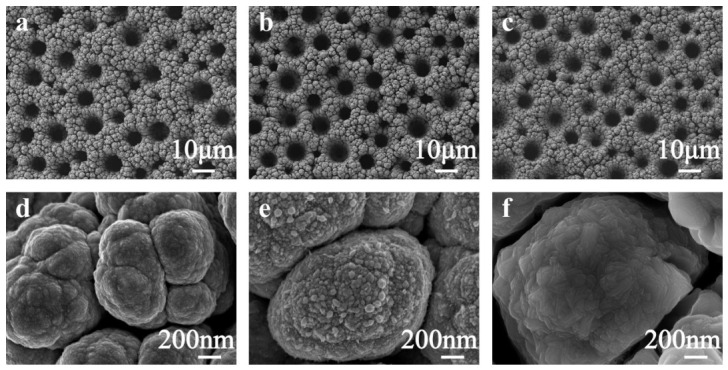
SEM images of (**a**,**d**) Ni; (**b**,**e**) Pt/Ni; and (**c**,**f**) PBA/Pt/Ni electrodes.

Following the DHBT electrodeposition process, the Ni skeleton presented a porous architecture (Figure 2a,d). Subsequent Pt electrodeposition resulted in a transformation of the smooth surface into a coarse morphology due to the formation of numerous nanocrystals, which is anticipated to enhance the specific surface area of the material, as depicted in Figure 2e. To illustrate the function of the porous Ni skeleton, Pt was directly electrodeposited on an Au-SPE using the same recipe as the Pt/Ni electrode. The Pt electrode showed a similar flat morphology to that of the Au-SPE (Figure S1b), with numerous nanoparticles anchored on the surface of substrate, which might exhibit an unsatisfactory electrochemical active area compared to the porous Pt/Ni electrode. To achieve a Pt/Ni film with an enhanced electrochemical active surface area, the synthesis parameters were systematically optimized, as shown in Figures S2 and S3. Finally, a NiFe PBA film was in situ synthesized on the Pt/Ni framework employing a sacrificial anode electrodeposition strategy. The progressive growth of the NiFe PBA layer was monitored via multi-cycle cyclic voltammetry. As the number of cycles increased, the redox peak currents associated with the PBA framework intensified consistently (Figure S4), confirming the successful and controllable in situ transformation of the sacrificial Ni surface into a dense PBA nanostructure. Under this controlled growth, the PBA particles appeared to be more compact, and even merged with other particles, which may provide a more efficient electron transport path (Figure 2f). To explore the distribution of different elements, EDS element mappings were conducted. In Figure S5, the Pt and Fe elements were distributed homogeneously, confirming the successful deposition of NiFe-based compounds on Pt/Ni skeletons.

**Figure 3:**
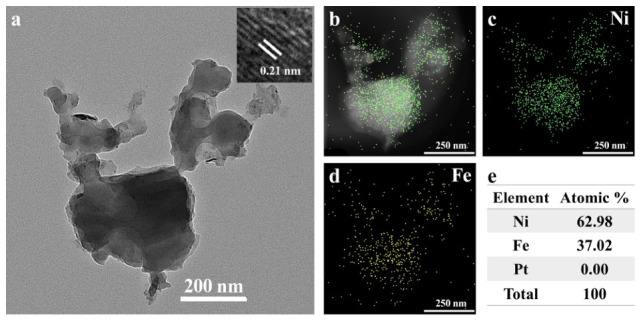
(**a**) TEM image, (**b**–**d**) HAADF-STEM images and element mapping, and (**e**) atomic ratio of NiFe PBA.

The microstructure of the PBA/Pt/Ni electrodes was further characterized by TEM. As displayed in Figure 3a, the PBA was in an irregular pointed shape, with a lattice fringe of 0.21 nm, which is assigned to the (422) plane of the NiFe PBA. Elemental analysis conducted on the NiFe PBA revealed that Ni and Fe elements were distributed uniformly among the nanoparticles, with an atomic ratio of about 63:37. This ratio closely aligns with the 2:1 stoichiometry for the NiFe PBA (Ni_2_Fe(CN)_6_), thereby verifying the successful formation of the intended NiFe PBA structure.

To verify the crystalline structures of products, XRD tests were carried out, and the XRD patterns of the fabricated Pt/Ni and PBA/Pt/Ni electrodes are presented in Figure S6. The XRD patterns of Pt/Ni mainly presented the characteristic peak of the Ni substrate, and the weak peak at about 40.1° was assigned to Pt. In the pattern of PBA/Pt/Ni electrodes, the diffraction peaks can be indexed to the (111), (220), and (222) crystallographic planes of the NiFe PBA (PDF#75-0037), further confirming the existence of the NiFe PBA, which was consistent with the TEM and EDS results.

**Figure 4:**
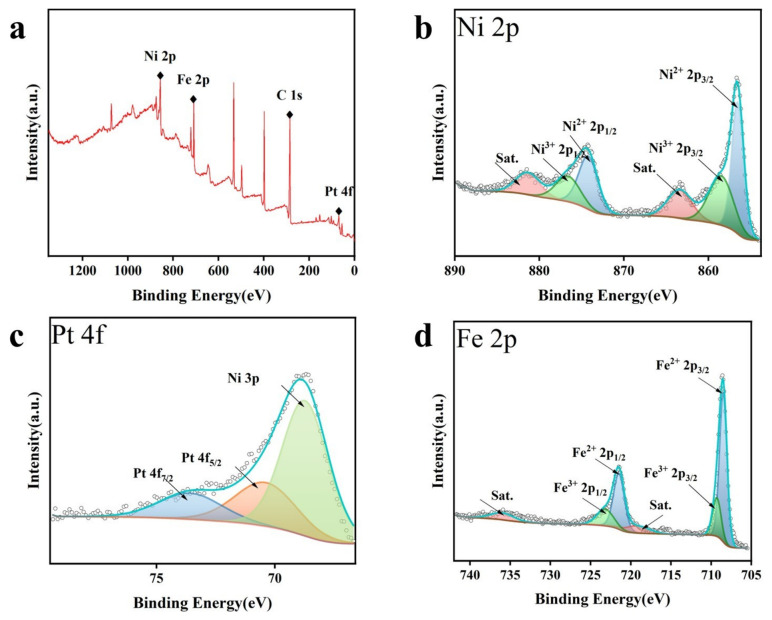
XPS spectra of PBA/Pt/Ni. (**a**) XPS survey spectrum; (**b**–**d**) high-resolution XPS spectra.

To explore the surface chemical compositions and oxidation states of the NiFe PBA/Pt/Ni/SPE composite electrodes, XPS analysis was carried out. The XPS survey spectrum presented in Figure 4a confirms the presence of Ni, Fe, and Pt elements [24]. After calibration against the C 1s peak, the binding energy (BE) of 874.2 eV and 856.6 eV for Ni 2p_1/2_ and Ni 2p_3/2_ can be indexed to Ni^2+^ species, and the peaks at 876.6 eV and 858.5 eV are assigned to the oxidized Ni^3+^ (Figure 4b). The Pt 4f spectra (Figure 4c) displayed a doublet at 70.4 eV and 73.6 eV, which are indexed to metallic Pt(0). An adjacent signal at 68.7 eV is likely derived from the Ni 3p orbital [25]. As for the Fe 2p spectra in Figure 4d, the fitted peaks at 708.5 and 721.5 eV can be attributed to 2p_3/2_ and 2p_1/2_ of Fe^2+^, while the peaks at 709.3 and 723.2 eV are assigned to Fe^3+^ [26]. In particular, the coexistence of multiple oxidation states—specifically the Ni^2+^/Ni^3+^ and Fe^2+^/Fe^3+^ redox couples—is instrumental in accelerating the inter-metal charge transfer through the cyanide bridges. This synergetic mixed-valence state not only provides a high density of electrocatalytic active sites but also optimizes the binding kinetics for hydrogen peroxide molecules, which fundamentally underpins the superior sensing performance discussed in the following section.

### 3.2. Electrochemical Characterization

**Figure 5:**
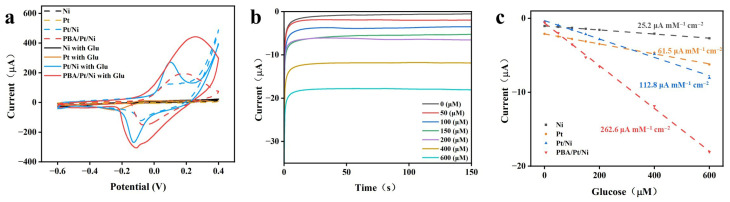
(**a**) The CV curves of GO_x_@SWCNTs immobilized Ni, Pt, Pt/Ni PBA/Pt/Ni electrodes with or without 300 μM glucose in PBS; (**b**) amperometric response of GO_x_@SWCNTs/PBA/Pt/Ni glucose biosensors at different glucose concentrations from 0 to 600 μM; (**c**) the fitting curves corresponding to Ni, Pt, Pt/Ni PBA/Pt/Ni electrodes.

Following a comprehensive analysis of the structure and composition of the PBA/Pt/Ni and other electrode materials, we subsequently assessed their electrocatalytic performance in glucose detection. For the fabrication of glucose biosensors, a composite enzyme membrane comprising glucose oxidase (GO_x_), single-walled carbon nanotubes (SWCNTs), and chitosan was synthesized and immobilized onto each of the prepared electrodes. The catalytic performance in glucose sensing was then assessed in PBS using cyclic voltammetry (CV). A concentration of 300 μM was strategically selected as it represents a typical physiological concentration of glucose in human sweat. As shown in Figure 5a, except for the Ni sensor, the other three kinds of GO_x_-modified electrodes displayed clearly increasing redox currents upon the addition of 300 μM of glucose. The GO_x_@SWCNTs/Pt/Ni sensor showed more distinct redox peaks compared to the GO_x_@SWCNTs/Pt counterparts, which is likely due to the high specific surface area of the porous architecture. The further improvement in the GO_x_@SWCNTs/PBA/Pt/Ni sensor is attributed to the exceptional electrocatalytic activity of the NiFe PBA with hydrogen peroxide [27]. The synergistic sensing mechanism can be elucidated by the following electrochemical cascade reactions (Equations (1)–(3)) [28]:

(1) $$\begin{array}{c} Glucose+O_{2}\overset{{\mathrm{GO}}_{x}}{\to }H_{2}O_{2}+\;Gluconic\;acid \end{array}$$

(2) $$\begin{array}{c} \mathrm{Ni}\left(\mathrm{II}\right)/\mathrm{Fe}\left(\mathrm{II}\right)+H_{2}O_{2}+2H^{+}\to \mathrm{Ni}\left(\mathrm{III}\right)/\mathrm{Fe}\left(\mathrm{III}\right)+2H_{2}O \end{array}$$

(3) $$\begin{array}{c} \mathrm{Ni}\left(\mathrm{III}\right)/\mathrm{Fe}\left(\mathrm{III}\right)+2e^{-}\to \mathrm{Ni}\left(\mathrm{II}\right)/\mathrm{Fe}\left(\mathrm{II}\right) \end{array}$$

In this catalytic cycle, glucose is first oxidized by GOx to generate H_2_O_2_ (Equation (1)). Subsequently, the NiFe PBA acts as an efficient electron mediator, where the redox transition between its different oxidation states facilitates the rapid reduction in H_2_O_2_ at the electrode interface (Equations (2) and (3)). This multi-step electron transfer process effectively amplifies the analytical signal, underpinning the superior sensitivity of the biosensor.

The working potential was optimized by evaluating the response to glucose across a range of potentials (−0.2 V to −0.05 V). A potential of −0.1 V was selected as it yielded the optimal balance between a high catalytic current and minimal background interference (Figure S7). To verify the sensing mechanism, the electrocatalytic activity of the PBA/Pt/Ni with H_2_O_2_ was investigated. As shown in Figure S8, the electrode exhibited a high sensitivity toward H_2_O_2_(800 μA mM^−1^·cm^−2^), confirming that the high sensitivity of the final biosensor is rooted in the superior reductive capacity of the NiFe PBA nanostructures with the enzymatically generated H_2_O_2_ intermediate. Figure 5b and Figure S9 show the biosensor responses of the GO_x_@SWCNT-immobilized electrodes at different glucose concentrations in PBS, with an applied potential of −0.1 V. The corresponding calibration curve of response current versus glucose concentration is illustrated in Figure 5c. The GO_x_@SWCNTs/PBA/Pt/Ni sensor exhibited impressive sensitivity (Y = −0.02889X − 0.638 R^2^ = 0.998, 262.6 μA mM^−1^·cm^−2^), an ultra-low detection limit of 1.45 μM (S/N = 3), and a rapid response time (less than 5 s), much better than the performance of GO_x_@SWCNTs/Pt/Ni (Y = −0.01241X − 0.313 R^2^ = 0.993, 112.8 μA mM^−1^·cm^−2^), GO_x_@SWCNTs/Pt (Y = −0.00676X − 2.107 R^2^ = 0.997, 61.5 μA mM^−1^·cm^−2^), and GO_x_@SWCNTs/Ni (Y = −0.00277X − 1.018 R^2^ = 0.998, 28.2 μA mM^−1^·cm^−2^). The synergistic role of the hierarchical support was further quantified by comparing the sensitivity of GO_x_@SWCNTs/Pt/Ni and GO_x_@SWCNTs/PBA/Pt/Ni. The integration of the NiFe PBA led to a 133% increase in sensitivity (from 112.8 to 262.6 μA mM^−1^·cm^−2^), confirming that the PBA nanostructures serve as the primary electrocatalytic engines. Furthermore, the platform demonstrated a negligible sensitivity to dissolved oxygen, with a baseline current deviation of <5% in N_2_-saturated environments (Figure S10).

To address the significance of the in situ synthesis of PBAs, the counterpart was prepared by drop-casting commercial PB onto Pt/Ni, and was named as the commercial PB/Pt/Ni electrode. The commercial PB nanoparticles, with an average size of ~30 nm, were prone to aggregating and blocking the porous structure (Figure S11). Therefore, the C_dl_ value of the commercial PB/Pt/Ni electrode was just about half that of the PBA/Pt/Ni electrode (Figure S12). This structural limitation and the poor interfacial contact were further quantified by EIS analysis in PBS (Figure S13), where the GO_x_@SWCNTs/PBA/Pt/Ni electrode demonstrated a 28% decrease in R_ct_ compared to the GO_x_@SWCNTs/commercial PB/Pt/Ni counterpart. Such a significant reduction in impedance highlights the effectiveness of the sacrificial anode electrodeposition in bypassing the high interfacial barriers typically found in physically assembled biosensing composites. Therefore, the glucose sensor GO_x_@SWCNTs/commercial PB/Pt/Ni merely achieved a sensitivity of 85.5 μA mM^−1^·cm^−2^ (Figure S14), inferior to that of the GO_x_@SWCNTs/PBA/Pt/Ni sensor or even the GO_x_@SWCNTs/Pt/Ni. The poor performance of the commercial PB/Pt/Ni electrode demonstrated that the strategy of the in situ synthesis of the NiFe PBA via the sacrificial anode electrodeposition method was beneficial in inhibiting the aggregation of PBA nanoparticles and enhancing the binding strength between the Pt/Ni substrate and the PBA, which then provided a more efficient electron transport path and greatly improved the electrochemical active area of the PBA/Pt/Ni electrode.

Furthermore, the proposed in situ synthesis strategy demonstrates superior cost-effectiveness for potential mass production compared to commercial PB routes. First, the method is highly time-efficient, integrating material synthesis and electrode modification into a single electrochemical step, which avoids the labor-intensive preparation of stable catalyst inks. Second, it requires low capital investment, as the electrodeposition process eliminates the need for expensive high-precision coating or printing equipment. Finally, the chemical anchoring of PBA nanostructures to the 3D framework results in a binder-free architecture, significantly streamlining post-processing by obviating the need for polymeric binders or prolonged drying cycles. These advantages, combined with the enhanced sensitivity, position this strategy as an economically viable route for the fabrication of next-generation wearable biosensors.

**Figure 6:**
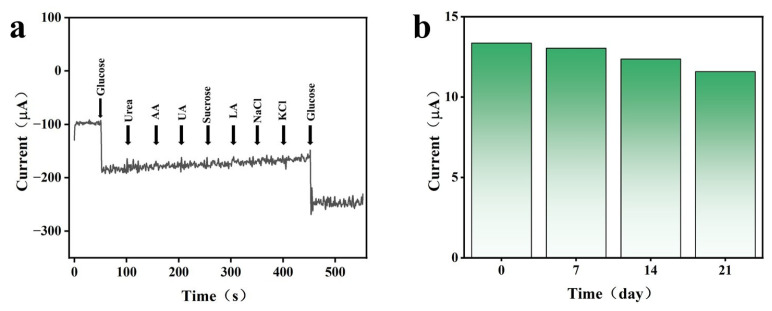
(**a**) Anti-interference performance of GO_x_@SWCNTs/PBA/Pt/Ni glucose sensor; (**b**) long-term stability of the biosensor over a period of 21 days.

To evaluate the practical application requirements, the anti-interference ability and long-term stability of the sensor are vital standards. The selectivity of the biosensor was evaluated at a working potential of −0.1 V using chronoamperometry. In the continuously stirred PBS solution, 100 µL of glucose (200 μM) and various common interfering substances (100 μM, including urea, ascorbic acid (AA), uric acid (UA), lactic acid (LA), KCl, and NaCl) were added successively. The results are shown in Figure 6a. The sensor showed a significant current response to glucose, while the response signals to various interferences were almost negligible. Notably, despite the theoretical capability of PBA materials for cation intercalation, the addition of NaCl and KCl induced no discernible amperometric response compared to the robust signal generated by glucose. After another injection of glucose, the current signal immediately increased, proving that the sensor has excellent selectivity for glucose.

In addition, the practicality of the sensor was further verified through long-term stability experiments. The sensor was stored at 4 °C and its current response to 300 μM glucose was measured initially and then once a week for three successive weeks. The response currents in the other three subsequent tests remained at 98%, 93%, and 87% of the initial response, respectively (Figure 6b). The sensor exhibited ~87% of its original current response after 21 days of storage/testing. Beyond storage stability, the operational stability of the sensor is paramount for continuous wearable monitoring. The device was subjected to a continuous amperometric test for 6 h at −0.1 V. The results (Figure S15) revealed remarkable stability, with less than 8% loss in the initial current. This performance is well-aligned with the requirements for wearable electronics, where single-use patches typically operate for less than 4 h and continuous monitoring devices target a 7–14-day lifespan. Both the 21-day storage stability (>85% retention) and the 6-h continuous operational stability (>92% retention) suggest that the PBA/Pt/Ni/Au-SPE architecture is robust enough to support a full wear cycle for semi-continuous monitoring.

To comprehensively evaluate the structural evolution of the sensor, SEM images of the electrode surface before and after prolonged testing were compared. As illustrated in Figure S16a, the as-prepared sensor features a pristine, well-defined micro/nano-porous nickel framework uniformly coated with a GO_x_/Nafion biofilm. Remarkably, even after continuous operational cycles, the SEM images of the ‘used’ sensor (Figure S16b) show that the underlying porous framework remains remarkably intact. There is no evidence of structural collapse or significant clogging of the pores, ensuring that the high specific surface area remains accessible for glucose diffusion and subsequent oxidation even after long-term use. Notably, the NiFe PBA nanostructures underneath the enzyme layer in Figure S16b remain firmly anchored to the Pt/Ni skeleton. This provides direct visual evidence that our in situ sacrificial anode electrodeposition creates a robust chemical-bond-level interface that can withstand the stresses of continuous operation. Significantly, this robust integration prevents the exfoliation of active materials and the detachment of the enzymatic membrane under sweat flow. The morphological integrity observed across Figure S16a,b is thus highly consistent with the electrochemical stability data (~87% sensitivity retention after 21 days), further confirming the durable nature of the proposed wearable sensor. From the above results, it can be concluded that the constructed GO_x_@SWCNTs/PBA/Pt/Ni biosensor presented excellent performance in anti-interference and stability and met the requirement for practical application.

### 3.3. Real Sweat Glucose Sensing

**Figure 7:**
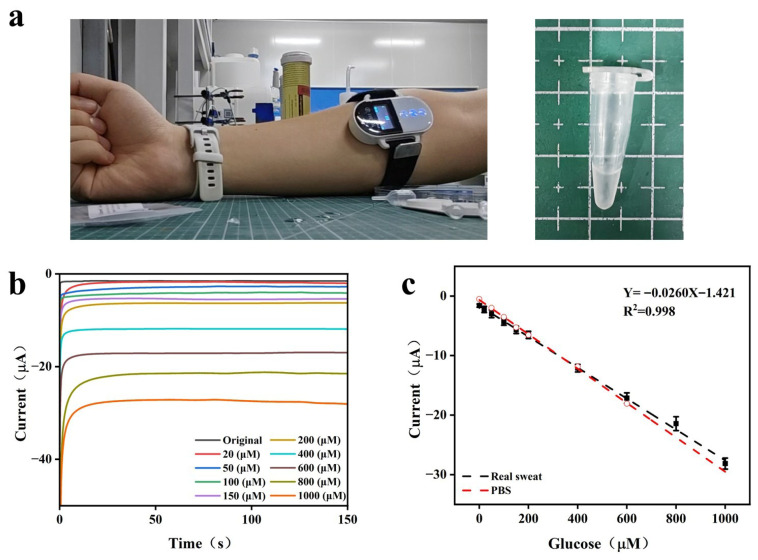
(**a**) Photograph of the wearable sweat sampler and the collected sweat; (**b**) amperometric response of the GO_x_@SWCNTs/PBA/Pt/Ni to successive additions of glucose in real sweat at −0.1 V; (**c**) glucose calibration curve obtained in sweat (red curve: measurement in PBS).

To validate the constructed biosensor for real-sample analysis, sweat was collected from the forearms of healthy adults using a wearable sweat sampler (Figure 7a), then diluted twice with PBS. The GO_x_@SWCNTs/NiFe PBA/Pt/Ni/Au-SPE glucose sensor was operated at a working potential of −0.1 V. The analytical performance in the sweat matrix was evaluated using the standard addition method, where known concentrations of glucose were incrementally spiked into the diluted sweat samples. As shown in Figure 7b,c, the sensor achieved a sensitivity of 236.4 μA mM^−1^·cm^−2^ and a detection limit of 2.19 μM (S/N = 3) in sweat. The wide linear range of 20–1000 μM totally covers the glucose concentration in human sweat. The matrix effect of authentic sweat was quantitatively evaluated by comparing the calibration curves in PBS and 1:2 diluted sweat samples. As illustrated in Figure 7c, the GO_x_@SWCNTs/PBA/Pt/Ni sensor exhibited a sensitivity of 236.4 μA mM^−1^·cm^−2^ (Y = −0.0260X − 1.421 R^2^ = 0.998) in the sweat matrix, which is 90.0% of the value obtained in pure PBS (262.6 μA mM^−1^·cm^−2^). The negligible sensitivity loss and the high correlation coefficient (R^2^ > 0.99) demonstrate the robust analytical performance of the platform. This minor deviation is well within the acceptable range for practical wearable sensing. These experimental findings demonstrated that the GO_x_@SWCNTs/PBA/Pt/Ni biosensor was capable of sensitive and accurate sweat glucose detection, underscoring its promising utility for wearable health monitoring.

The performance of the GO_x_@SWCNTs/PBA/Pt/Ni biosensor and some previously reported enzymatic glucose sensors utilizing noble metals or Prussian blue materials are summarized in Table 1 and Table 2. Evidently, the GO_x_@SWCNTs/PBA/Pt/Ni biosensor exhibits a relatively low detection limit and a high sensitivity. These advantages can be attributed to the distinctive structural design. The three-dimensional porous Ni framework offers a large specific surface area that facilitates the homogeneous distribution of Pt and NiFe PBAs. Simultaneously, the tight interaction between the Pt/Ni skeleton and the NiFe PBA might boost the electron transfer speed and further improve the electrochemical activity, thereby jointly achieving a significant improvement in sensor performance. However, while the GO_x_@SWCNTs/PBA/Pt/Ni sensor exhibits exceptional analytical performance, several caveats regarding its clinical utility must be addressed. It is essential to recognize that sweat glucose levels are profoundly influenced by dynamic physiological variables, including localized perspiration rates and individual metabolic heterogeneity, which present significant challenges to maintaining clinical consistency across diverse populations. Consequently, the current platform is positioned as a sophisticated tool for trend monitoring and personal wellness tracking rather than a definitive replacement for gold-standard blood glucose diagnostics. Extensive clinical investigations remain imperative to further elucidate the complex, non-linear correlations between sweat and systemic blood glucose under varying physiological conditions.

## 4. Conclusions

In this work, a high-performance sweat glucose sensor was developed through a three-step electrochemical deposition approach, resulting in a composite electrode architecture based on NiFe PBA/Pt/Ni/Au-SPE. Detailed structural characterization has confirmed the successful formation of NiFe PBA nanocrystals with uniform distribution. The constructed GO_x_@SWCNTs/NiFe PBA/Pt/Ni/Au-SPE biosensor exhibited outstanding sensing performance in PBS, demonstrating a high sensitivity of 262.6 μA mM^−1^·cm^−2^, a low detection limit of 1.45 μM, and a broad linear detection range from 20 to 600 μM. Furthermore, the practical utility of the platform was validated through successful application in sweat analysis. The enhanced sensitivity of the sensor is attributed to the porous structure afforded by the Pt/Ni matrix. Furthermore, the in situ synthesis of the NiFe PBA was achieved via a sacrificial anode electrodeposition approach, and effectively suppressed nanoparticle agglomeration and strengthened the adhesion between the PBA layer and the Pt/Ni substrate, leading to a synergistic improvement in sensor performance. Overall, this work has developed a novel material and structural design for advanced biosensing applications, revealing its promising potential for integration into a non-invasive wearable glucose monitoring system based on sweat analysis.

## Figures and Tables

**Table 1:** Noble metal-based enzymatic glucose sensors.

Sensor Material	Linear Range (μM)	Response Time (s)	LOD (μM)	Sensitivity (μA mM^−1^·cm^−2^)	Refs.
LSG/PBSE/PtNPs/GO_x_/nafion	5–3200	5	2.57	12.64	[29]
GO_x_@SWCNT/PtPd/Au-SPE	20–410	/	16	112	[30]
GO_x_/AuNP/WS2/PEDOT-PSS/ITO	740–440,610	/	1	13.1	[31]
GO_x_@Au NPs/graphene	43–261	/	8.9	64	[32]
GO_x_/BSA/GNPs-Pb NWs/Pt	5–2200	<5	2	135.5	[33]
GO_x_/PEDOT/Pt	200–8000	2–5	40	15.2	[34]
GO_x_@SWCNTs/NiFe PBA/Pt/Ni/Au-SPE	20–1000	<5	2.19	236.4	This work

**Table 2:** Prussian blue-based enzymatic glucose sensors.

Sensor Material	Linear Range (μM)	Response Time (s)	LOD (μM)	Sensitivity (μA mM^−1^·cm^−2^)	Refs.
GO_x_/Co Fe PBA@LIG	25–2000	/	25	31.28	[35]
GO_x_/PB/MWNTs-GEC	250–1300	10	7.5	15	[36]
GO_x_/PB/PEDOT:PSS	1–243	/	0.85	340.1	[37]
GO_x_/PB/PC	30–400	/	30	218.78	[18]
GO_x_/PB/AuNPs	25–2000	2	1.62	40.41	[38]
GO_x_/PB-RGO	300–2100	/	7.94	27.78	[39]
GO_x_@SWCNTs/NiFe PBA/Pt/Ni/Au-SPE	20–1000	<5	2.19	236.4	This work

## Data Availability

The original contributions presented in this study are included in the article. Further inquiries can be directed to the corresponding author.

## Supplementary Materials

The following supporting information can be downloaded at: https://www.mdpi.com/article/10.3390/s26092908/s1, Figure S1. SEM image: (a,c) Au-SPE; (b,d) Pt electrodeposited on Au-SPE; Figure S2. (a–d) SEM images of the Pt/Ni electrode prepared under different deposition potentials with a deposition time of 40 s. (e) Double-layer capacitance of the electrode prepared under different deposition potentials (determined by CV test of non-faradaic region in 0.5M Na_2_SO_4_); Figure S3. (a–d) The SEM images Pt/Ni electrode electrodeposited for different time, deposition voltage was −3V. (e) double-layer capacitance of electrode electrodeposited for different time (determined by CV test of non-faradaic region in 0.5M Na_2_SO_4_); Figure S4. Electrochemical monitoring of NiFe PBA; Figure S5. EDS mapping of the PBA/Pt/Ni; Figure S6. XRD patterns Pt/Ni and PBA/Pt/Ni electrodes; Figure S7. Optimization of the applied working potential: Amperometric responses of the GO_x_@SWCNTs/PBA/Pt/Ni sensor at different applied potentials from −0.2 V to −0.05 V; Figure S8. The chronoamperometric response and the corresponding calibration curve of the PBA/Pt/Ni electrode toward H_2_O_2_; Figure S9. Amperometric response of (a) GO_x_@SWCNTs/Ni; (b) GO_x_@SWCNTs/Pt; (c) GO_x_@SWCNTs/Pt/Ni; glucose biosensors at different glucose concentrations from 0 to 600 μM; Figure S10. The baseline stability in nitrogen-saturated (N_2_) versus air-saturated PBS (pH 7.4); Figure S11. SEM images of commercial PB drop-casting on Pt/Ni electrode; Figure S12. (a) The CV test of non-faradaic region in 0.5 M H2SO4: (a) commercial PB/Pt/Ni; (b) PBA/Pt/Ni and (c) plots of the current density at 0.14V vs the scan rate; Figure S13. Nyquist plots of the GO_x_@SWCNTs/PBA/Pt/Ni and GO_x_@SWCNTs/commercial PB/Pt/Ni sensors measured at −0.1 V in PBS; Figure S14. (a) The GO_x_@SWCNTs/commercial PB/Pt/Ni performance diagram, (a) the current response when different concentrations of glucose are added at a voltage of −0.1V in PBS; (b) The corresponding linear calibration curve of current vs. glucose concentration; Figure S15. Current response recorded continuously for 6 hours at −0.1 V in PBS solution containing 300 μM glucose; Figure S16. SEM images of the electrode surface (a) before and (b) after the stability test.
