# Supplementary material for: A High-Sensitivity Sweat Glucose Biosensor Enabled by an In Situ Grown NiFe PBA on Porous Pt/Ni/Au-SPE
Source: Sensors (Basel). 2026 May 6;26(9):2908. doi: 10.3390/s26092908 (PMC13165853; doi:10.3390/s26092908)
Supplement: Supplementary file 1 [file sensors-26-02908-s001.zip › sensors-4266937-supplementary.docx]

**High-sensitivity sweat glucose biosensor enabled by in situ grown NiFe PBA on porous Pt/Ni/Au-SPE**

Huajie Shu^†^, Qinglin Liu^†^, Qianhui Wei, Changhui Mao, Feng Wei^*^, Hailing Tu

^†^ These authors contributed equally to this work.


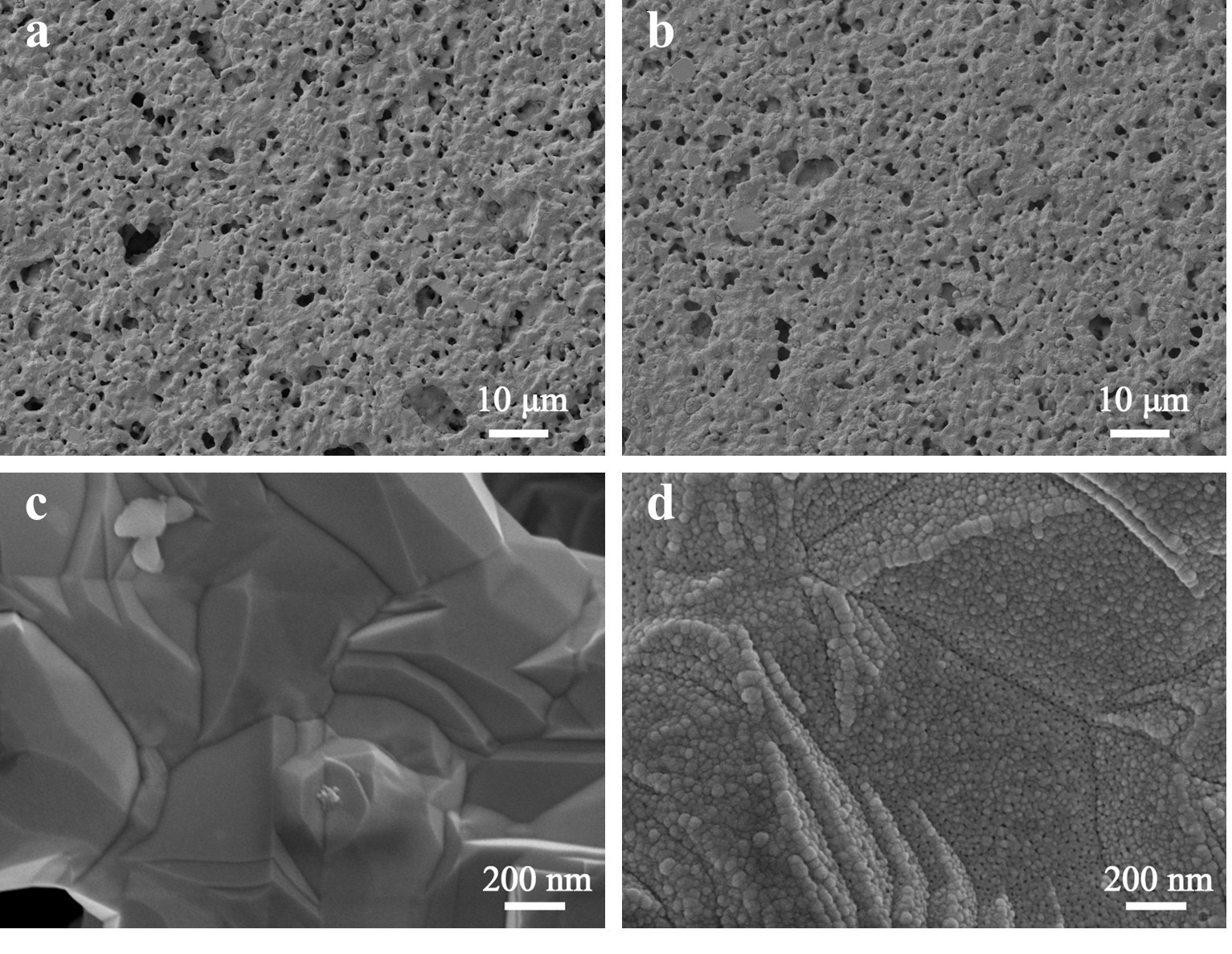


Figure S1. SEM image: (a, c) Au-SPE; (b, d) Pt electrodeposited on Au-SPE.


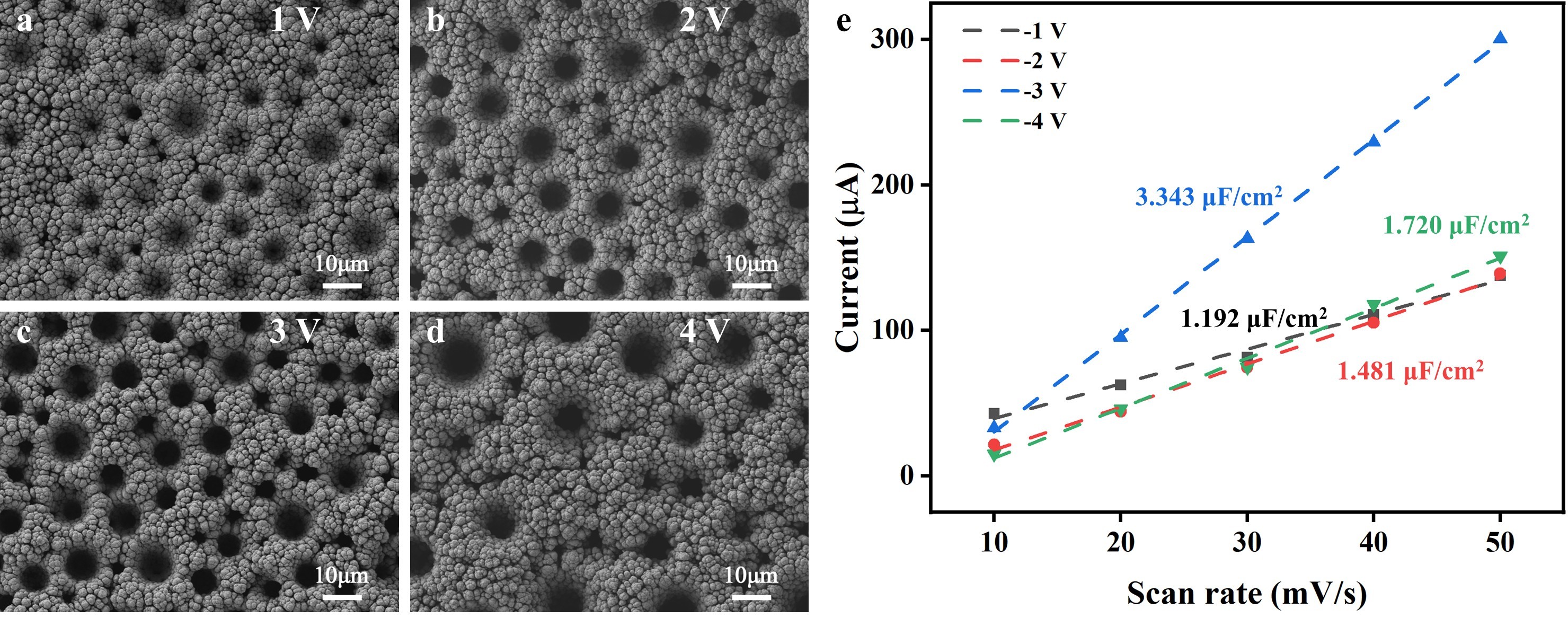


Figure S2. (a–d) SEM images of the Pt/Ni electrode prepared under different deposition potentials with a deposition time of 40 s. (e) Double-layer capacitance of the electrode prepared under different deposition potentials (determined by CV test of non-faradaic region in 0.5M Na_2_SO_4_).

From the SEM images, it could be observed that the Pt/Ni electrode prepared under -3V exhibited the most clearly porous structure. This phenomenon was probably because when the deposition potential was more positive, the hydrogen produced more slowly, the porous structure was hard to form, but if the deposition potential was more negetive, the Pt^4+^ deposited much faster and blocked the pore. Therefore, the Pt/Ni electrode prepared under -3V also ehibited the highest C_dl_ value and possesses the largest electrochemically active area.


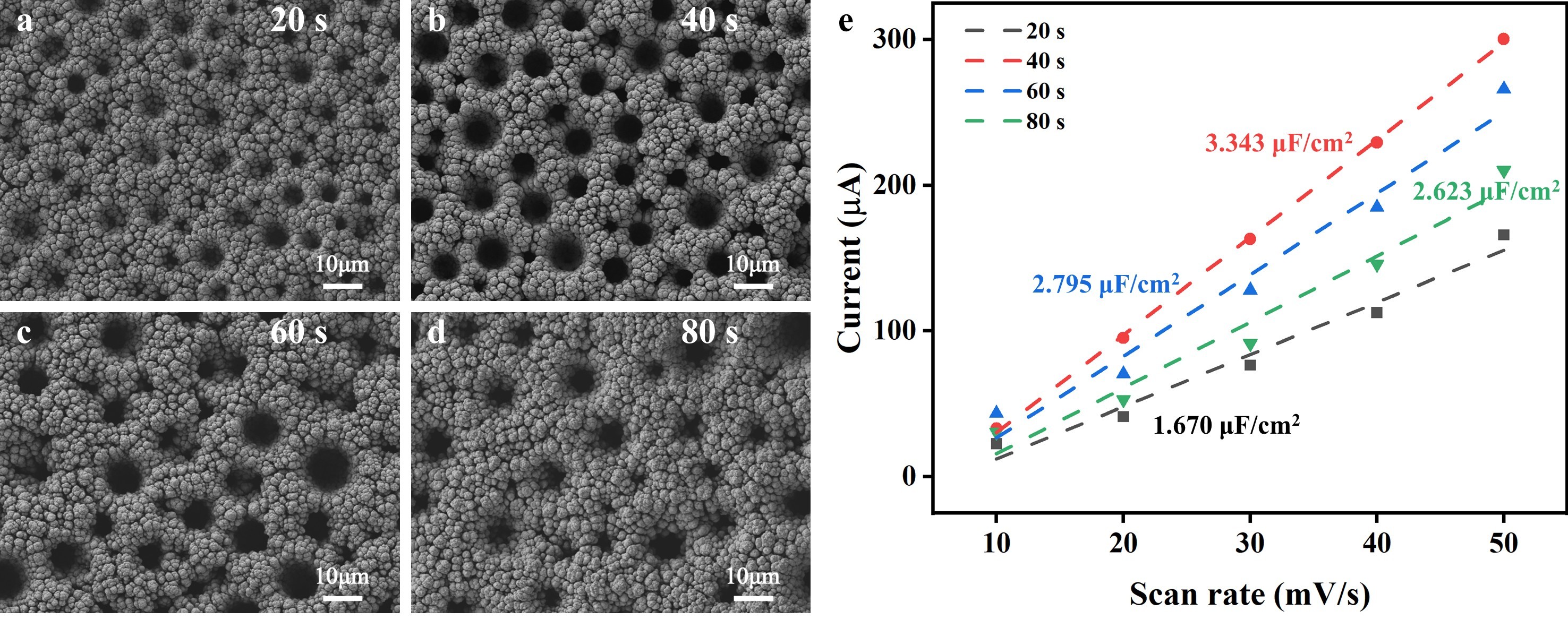


Figure S3. (a-d) The SEM images Pt/Ni electrode electrodeposited for different time, deposition voltage was -3V. (e) double-layer capacitance of electrode electrodeposited for different time (determined by CV test of non-faradaic region in 0.5M Na_2_SO_4_).

From the SEM images, it could be observed that the Pt/Ni electrode electrodeposited for 40s exhibited the most clearly porous structure. This phenomenon was probably because when the deposition reaction was conducted for longer time, more Pt^4+^ deposited and blocked the pore. Hence, the Pt/Ni electrode electrodeposited for 40s also showed the highest C_dl_ value possesses the largest electrochemically active area.


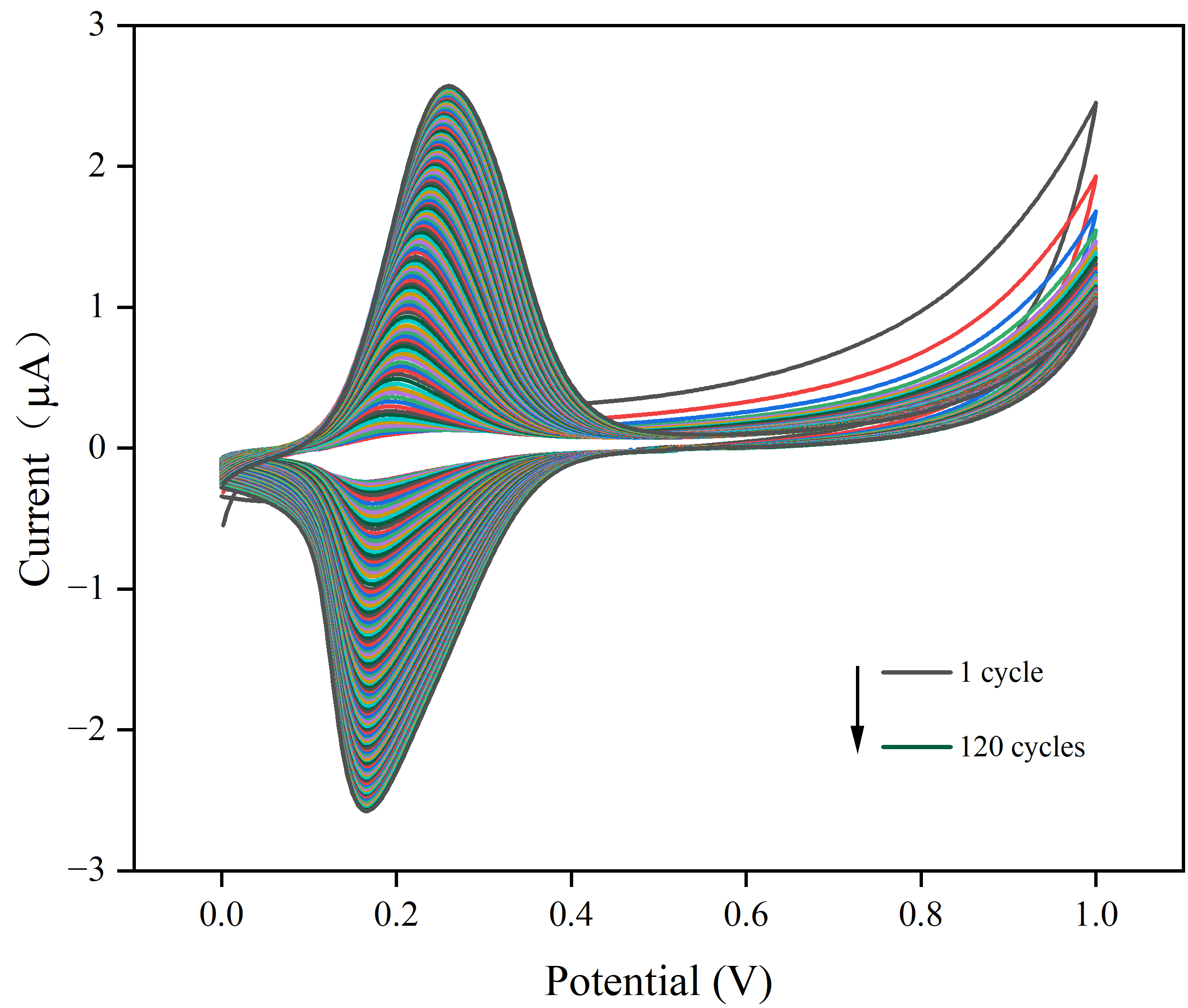


Figure S4. Electrochemical monitoring of NiFe PBA.


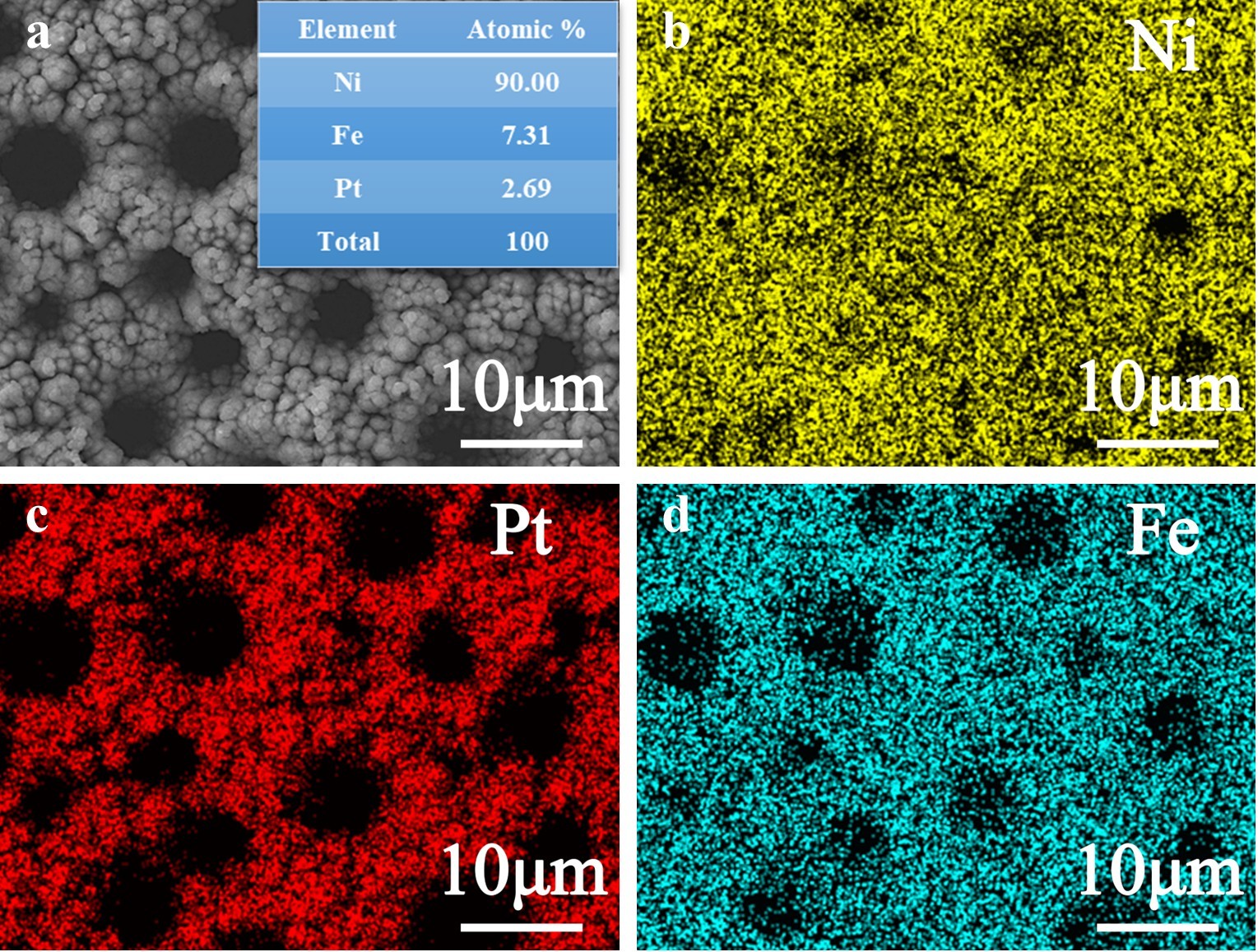


Figure S5. EDS mapping of the PBA/Pt/Ni.


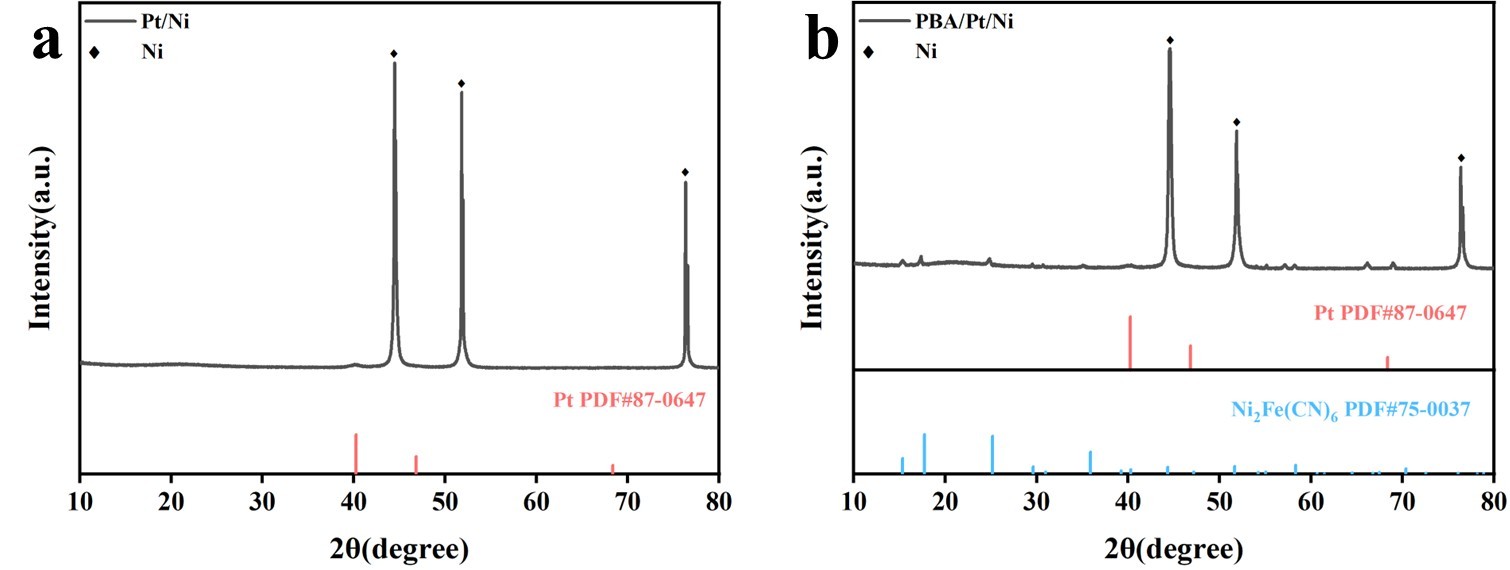


Figure S6. XRD patterns Pt/Ni and PBA/Pt/Ni electrodes.


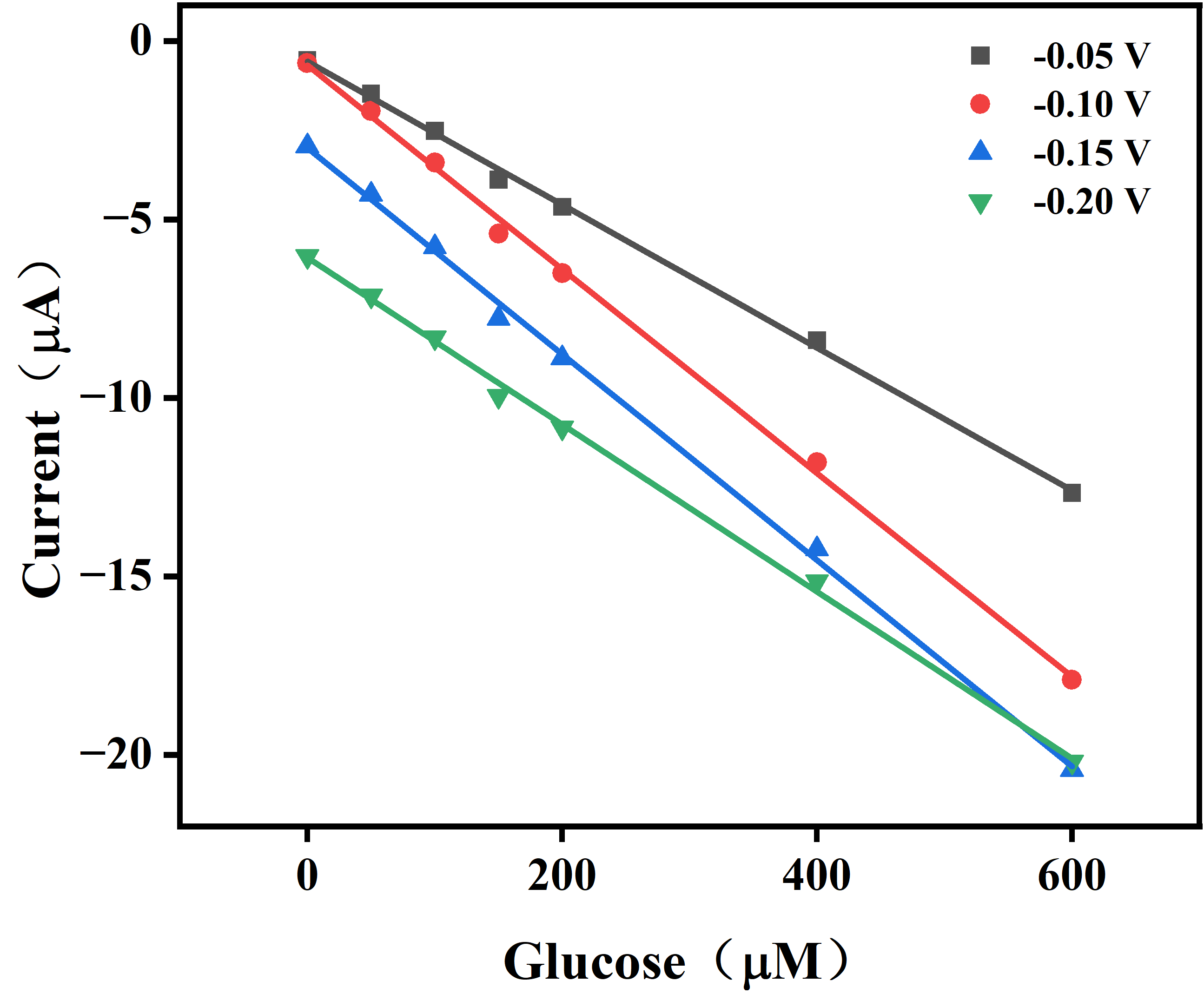


Figure S7. Optimization of the applied working potential: Amperometric responses of the GO_x_@SWCNTs/PBA/Pt/Ni sensor at different applied potentials from -0.2 V to -0.05 V.


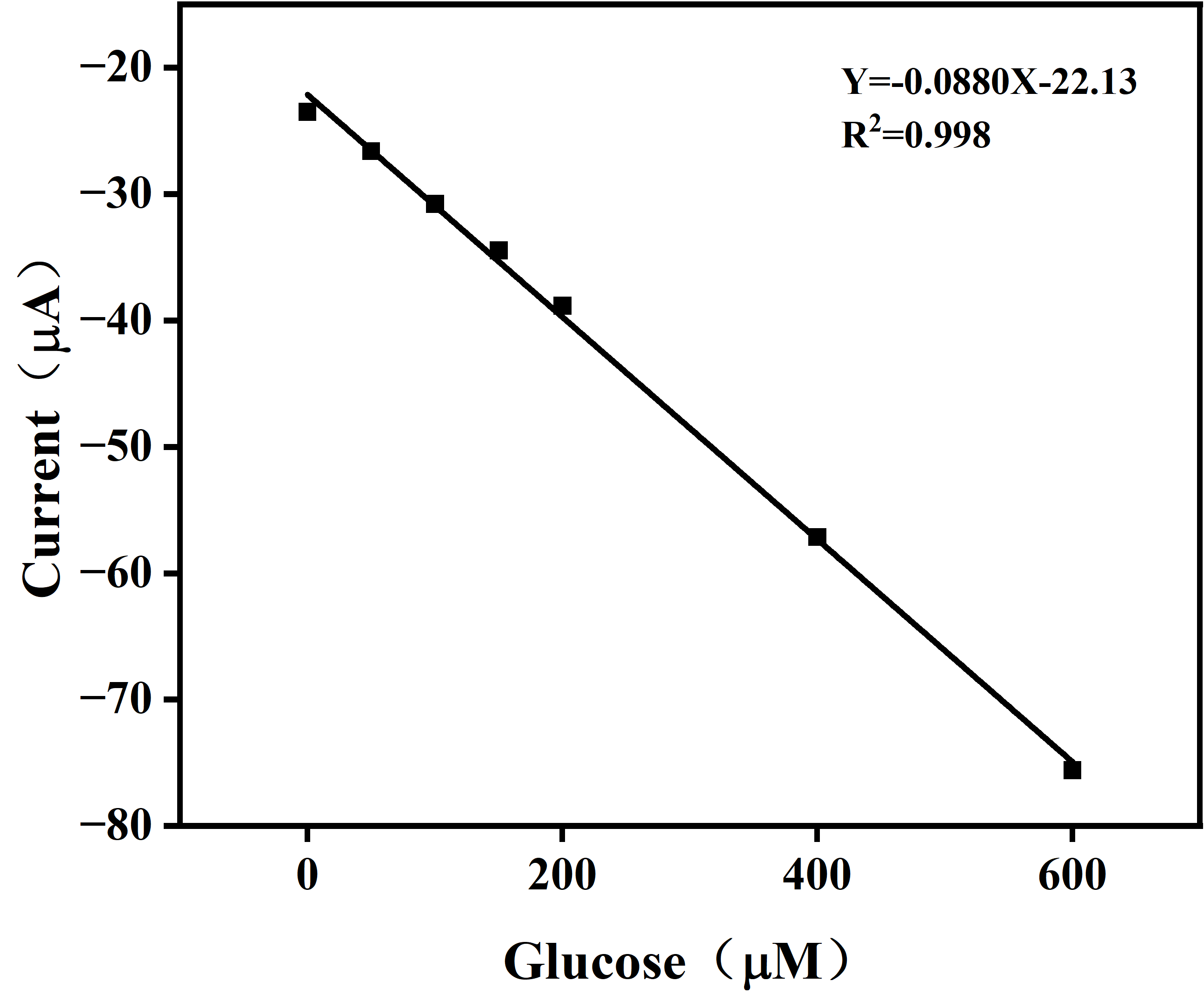


Figure S8. The chronoamperometric response and the corresponding calibration curve of the PBA/Pt/Ni electrode toward H_2_O_2_.


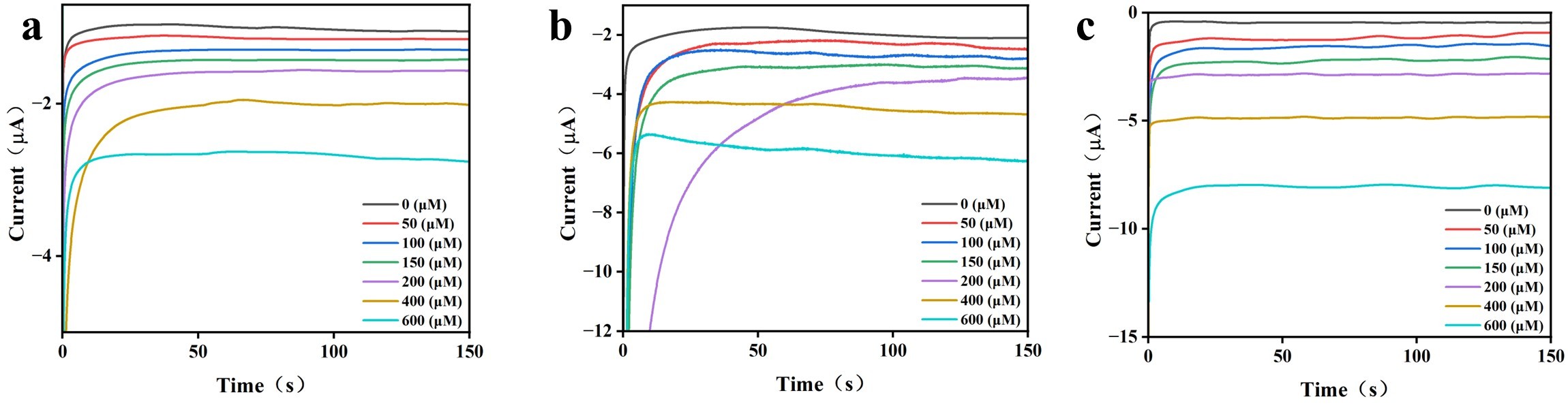


Figure S9. Amperometric response of (a) GO_x_@SWCNTs/Ni; (b) GO_x_@SWCNTs/Pt; (c) GO_x_@SWCNTs/Pt/Ni; glucose biosensors at different glucose concentrations from 0 to 600 μM.


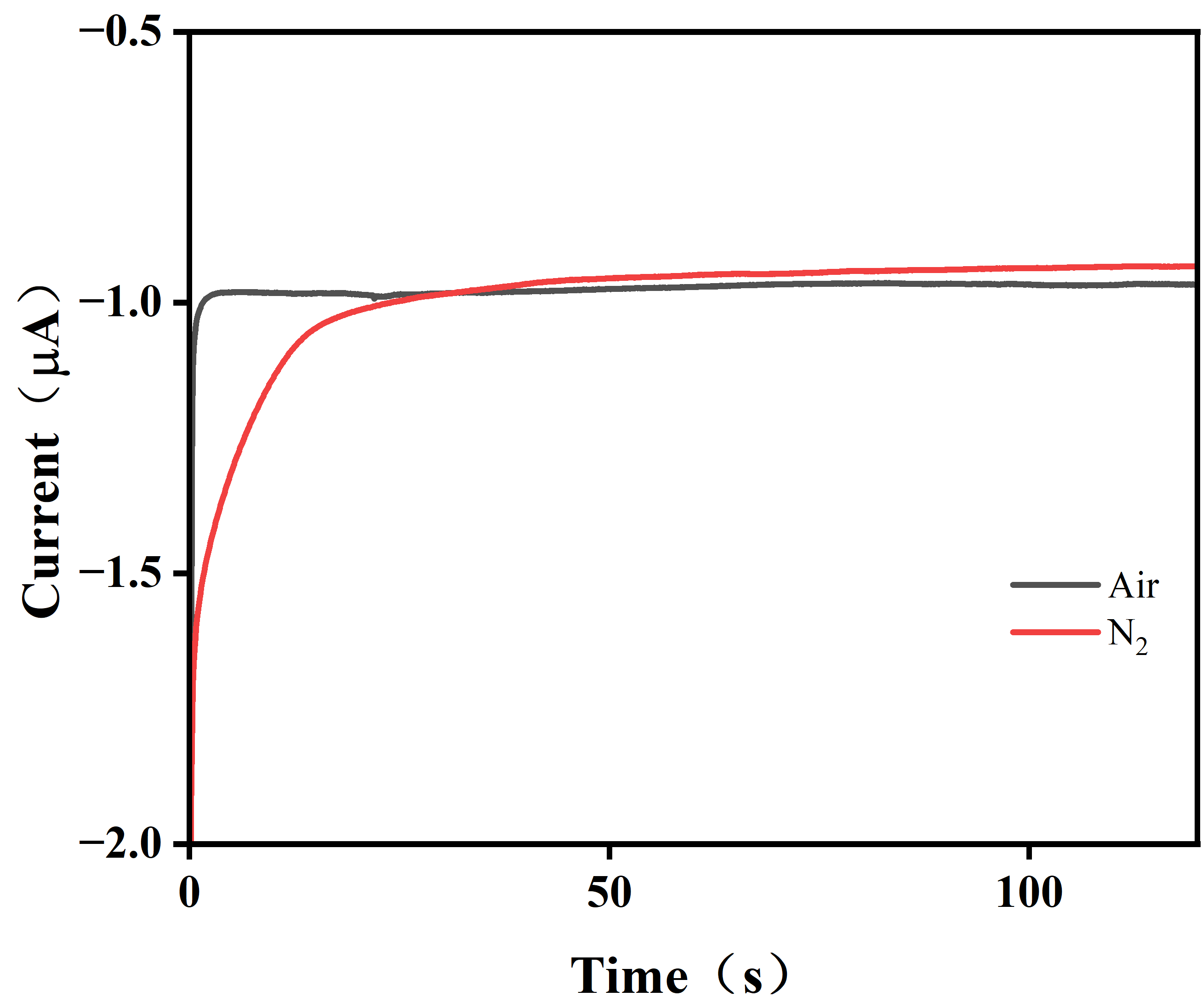


Figure S10. The baseline stability in nitrogen-saturated (N_2_) versus air-saturated PBS (pH 7.4).


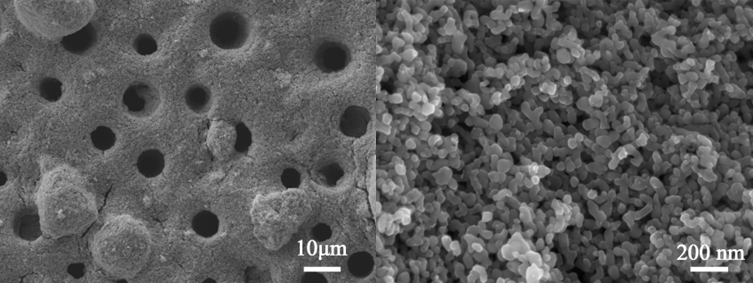


Figure S11. SEM images of commercial PB drop-casting on Pt/Ni electrode.


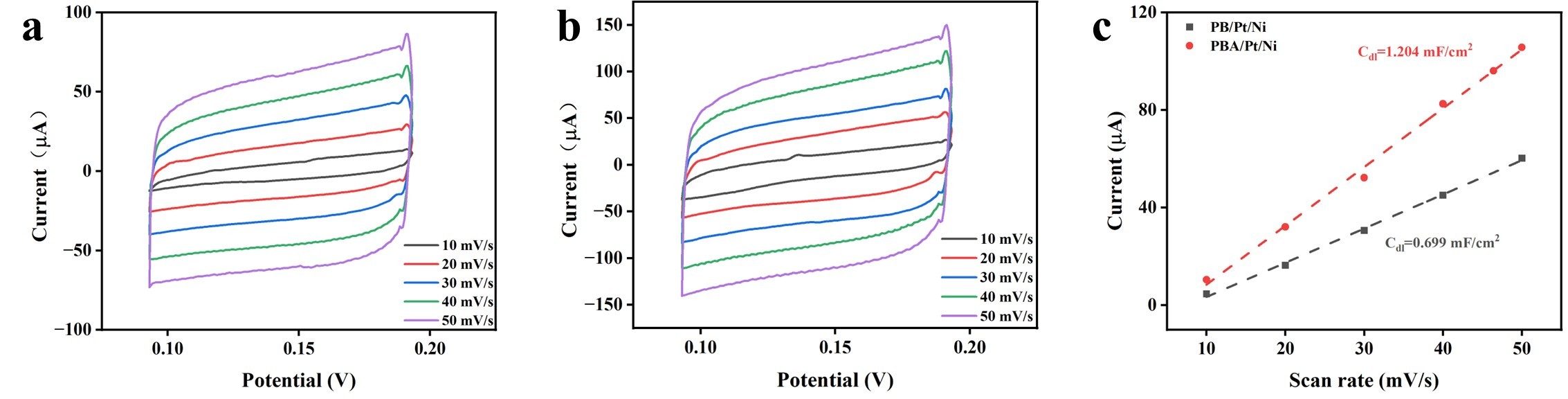


Figure S12. (a) The CV test of non-faradaic region in 0.5 M H_2_SO_4_: (a) commercial PB/Pt/Ni; (b) PBA/Pt/Ni and (c) plots of the current density at 0.14V *vs* the scan rate.


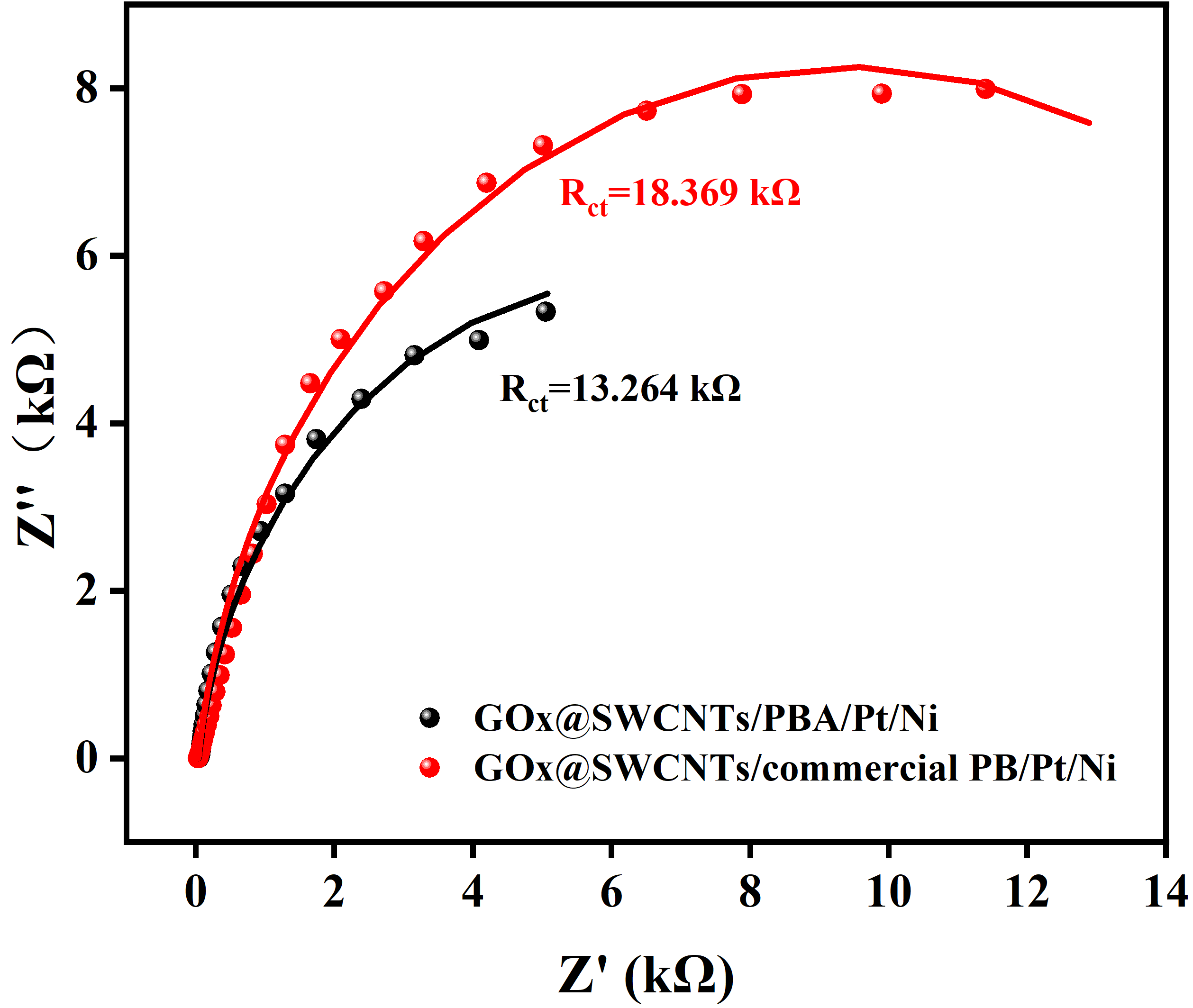


Figure S13. Nyquist plots of the GO_x_@SWCNTs/PBA/Pt/Ni and GO_x_@SWCNTs/commercial PB/Pt/Ni sensors measured at -0.1 V in PBS.


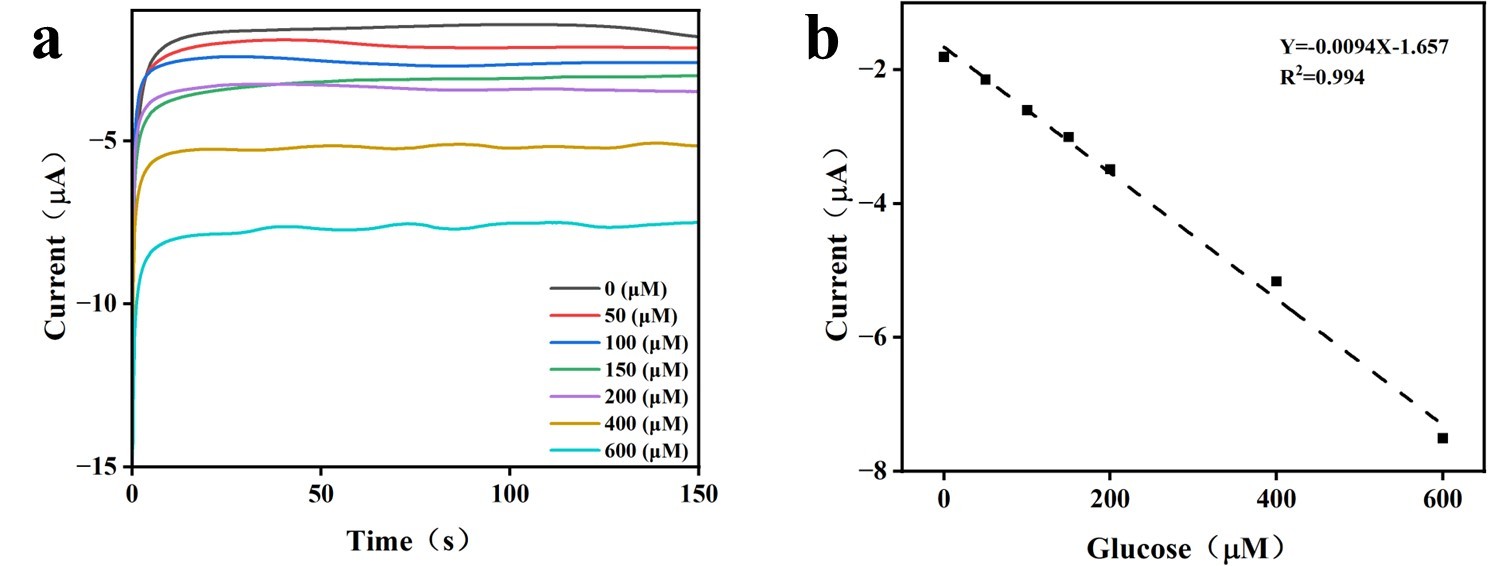


Figure S14. (a) The GO_x_@SWCNTs/commercial PB/Pt/Ni performance diagram, (a) the current response when different concentrations of glucose are added at a voltage of -0.1V in PBS; (b) The corresponding linear calibration curve of current vs. glucose concentration.


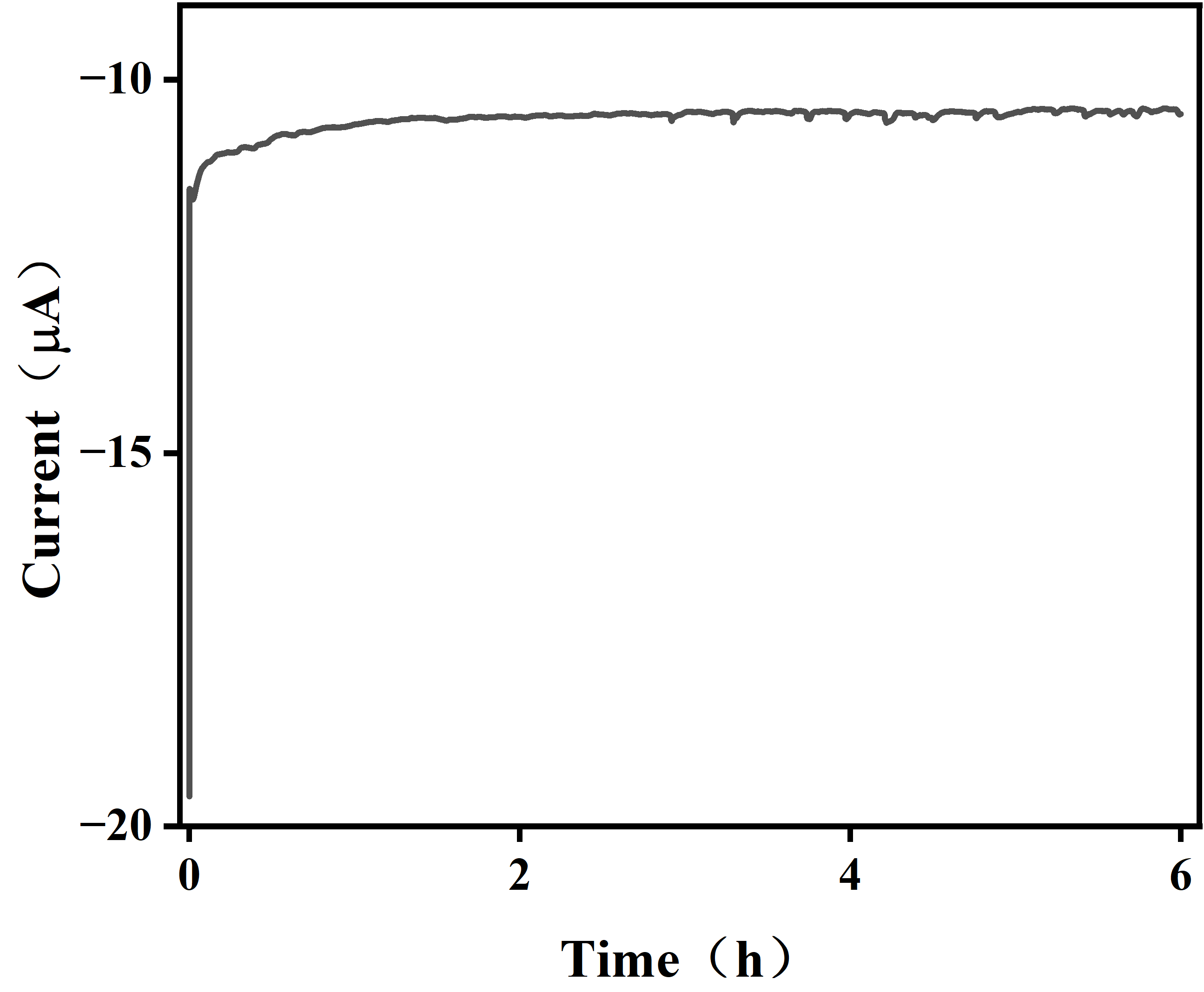


Figure S15. Current response recorded continuously for 6 hours at -0.1 V in PBS solution containing 300 μM glucose.


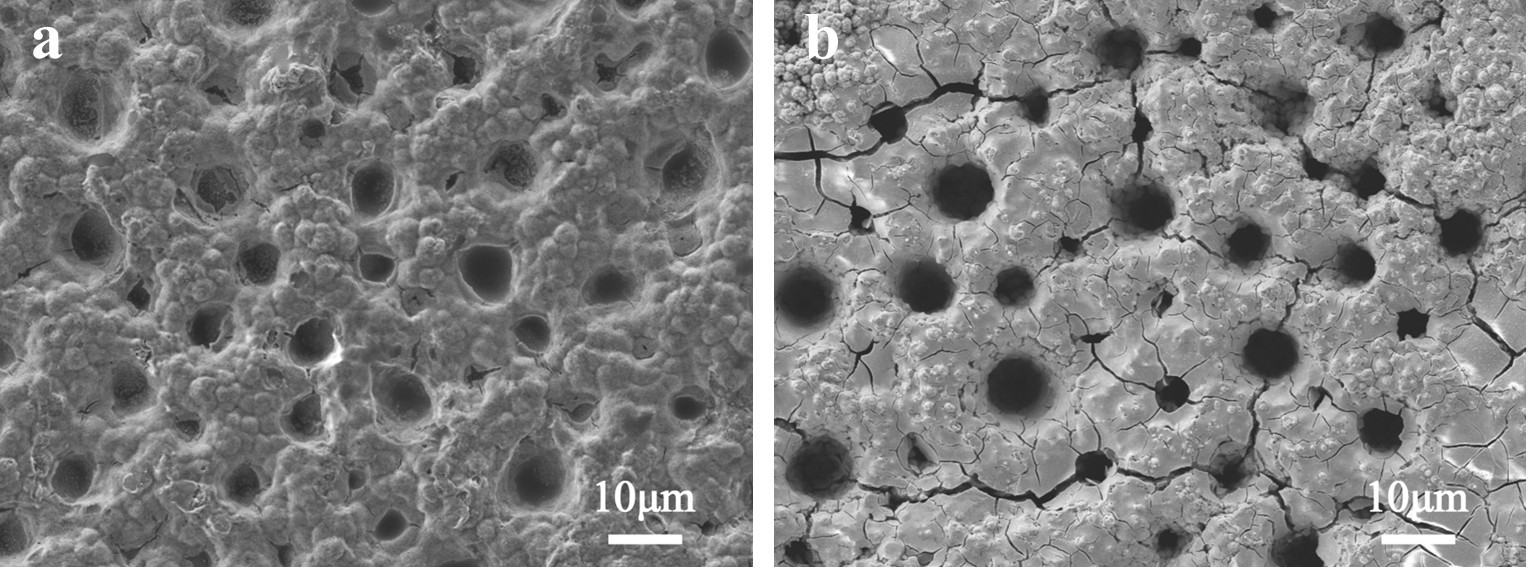


Figure S16. SEM images of the electrode surface (a) before and (b) after the stability test.
